# Evidence-based veterinary medicine at 20 – a commentary on historical, philosophical, practical, and ethical aspects

**DOI:** 10.18849/ve.v10i3.710

**Published:** 2025-07-09

**Authors:** David Mills+, Michael Reiss+, Madeleine Campbell+

**Keywords:** EPISTEMOLOGY, ETHICS, EVIDENCE-BASED VETERINARY MEDICINE, EVOLUTION, FUTURE, HISTORY

## Abstract

Evidence-based veterinary medicine (EBVM) has pervaded the veterinary profession in the last two decades including academic and regulatory arenas as well as practising veterinary surgeons. EBVM grew from its older human sister, evidence-based medicine (EBM) and in doing so shares EBM’s central tenets of how best to do medicine. However, EBM has by no means been universally accepted; it has attracted fierce criticism and undergone several revisions since inception. In this article, we trace history of both movements and critically examine the philosophical basis of EBM/EBVM being better. We also assess the practicality of EBVM and examine ethical aspects of its use. With knowledge of EBM’s evolution, possible ways forward for EBVM are suggested that attempt to avoid EBM’s historical setbacks and improve individual veterinary patient care under EBVM.

## One sentence summary

The authors present a commentary on the historical, philosophical, practical, and ethical aspects of EBVM, identifying inherent and applied challenges before offering possible solutions to better shape its future applicability to veterinary medicine.

## Introduction

Publication of the first textbook dedicated to EBVM, the* Handbook of Evidence-based Veterinary Medicine* by Cockcroft & Holmes (2003), formalised the EBVM discipline just over 20 years ago although the concept had been briefly mentioned in other texts around that time (Bonnett, 1998; Polzin et al., 2000). Since then, EBVM has gained traction in academic and regulatory arenas, and within the general professional narrative, of veterinary medicine. In the UK, the Royal College of Veterinary Surgeons (RCVS) has stated that, when available, its adoption is required to be considered fit to practise (Jorge & Pfeiffer, 2012). The American Veterinary Medical Association (AVMA) and Canadian (CVMA) Veterinary Medical Association require veterinarians to practise by applying scientific knowledge, and their position statements require decisions to be evidence-based (AVMA, 2024; CVMA, 2020). In the Competency-Based Veterinary Education (CBVE) framework, EBVM is cited as central to clinical reasoning and decision-making (CBVE, 2024). Adoption of CBVE and other similar iterations continues apace in UK and North American universities. Whilst uptake of application by general veterinary practitioners has been less rapid, awareness of EBVM has increased in the last two decades, possibly partly due to increased exposure during undergraduate teaching (Vandeweerd et al., 2012a; Nielsen et al., 2015).

EBVM protagonists argue that it is a superior way of performing veterinary medicine, as it is more reliable, objective and delivers the best outcomes for the individual patient. It is therefore the most ethically sound approach to veterinary medicine (Ramey & Rollin, 2001; Jorge & Pfeiffer, 2012).

However, the practicality, epistemology, and ethical implications of EBVM have not been subject to any detailed pragmatic or philosophical critique. This article, will look at the historical beginnings and elopment of EBM and EBVM, the philosophical underpinning of EBM/EBVM’s central objectivity claim, examine practical problems with its application, and briefly consider the ethical arguments for using it. We argue that EBM has evolved considerably since its inception in the 1990s, partly due to criticisms about its application to the individual patient and the usability of its methodology. EBVM as a younger relative is not as far along its development journey. Expedient lessons that avoid the missteps of EBM’s history can help EBVM adapt to better serve individual veterinary patients.

We offer our opinions and perspectives from a background of clinical veterinary practice at both first opinion and specialist levels and longstanding interest in medical and scientific epistemology and applied ethics.

## A brief history of evidence-based medicine

Evidence-based medicine has its philosophical roots in Napoleonic Paris of the early nineteenth century (Sackett et al., 1996). Alexandre Louis and others proposed a movement of médicine d’observation in which physicians should not rely on speculation but should rely on large series observations and derive numerical therapy from those results (Vandenbroucke, 1996). It took some inspiration from empirical epidemiological work happening in Britain at the same time, such as John Snow’s elucidation of water pump cholera outbreaks in London (Sackett et al., 1996). Whilst this movement foundered on a combination of a lack of contextual evidence with which to illustrate its effectiveness and strong contemporary resistance, it was to re-emerge in the mid-20^th^ century in Europe in more or less the same basic form. The first scientific work truly within the EBM sphere – although the term had not yet been coined – was produced in 1955 (Chalmers et al., 1955). This work, a controlled factorial trial by Tom Chalmers, for the first time explicitly integrated clinical epidemiology, meta-analysis, and individual patient care outcomes. This was followed by an exposition of clinical epidemiology and a vision for medical practice study by Alvan Feinstein (Feinstein, 1963). A series of lectures by Archie Cochrane in 1972 argued that too much healthcare was being practised on the basis of unknown or flimsy evidence, endangering patients, harming individuals and populations, wasting resources, and failing patients. His proposal was that treatments should be evaluated in an unbiased, objective way, properly audited, by a combination of individual clinicians and clinical epidemiological researchers; he saw this as the only method to satisfy the fundamental medical ethics of doing no harm, doing good, and targeting resources effectively (Cochrane, 1972).

The EBM approach came to prominence largely due to the McMaster University group of David Sackett, Gordan Guyatt, Brian Haynes, and Peter Tugwell in the early 1990s who, along with similar-minded academics, went on to form the Evidence Based Medicine Working Group. They formulated a new approach to undergraduate medical education (‘scientific medicine’), instigating curricula founded on problem-based learning, with basic science, epidemiology, public health, and statistics being taught as integral parts of the course (Zimerman, 2013; Smith & Rennie, 2014). They took as their base clinical epidemiology and looked to expand the epidemiological features of disease into medical decision-making (Sackett et al., 1996). Clinical epidemiology held that critical appraisal skills in healthcare workers, were as fundamentally required as diagnostic and technical skills (Sackett et al., 1996; Liberati & Vineis, 2004). Critical appraisal was defined as the sceptical assessment of research underpinning clinical interventions and proved a turning point in allowing delineation of opinion expert-based medicine from objective scientific evidence (Sackett, 1981). A significant product of clinical epidemiology was the highlighting of the poor methodological quality underlying much evidence related to diagnosis, treatment and prognosis. A series of publications, demonstrations, and articles outlined this new theory of medicine and quickly gained traction within the medical and scientific communities.

The term “evidence-based medicine” was first used in a landmark 1992 article by the Evidence Based Medicine Working Group which opined that all diagnosis, prognosis, and therapy should be based on qualitative data derived from clinical epidemiology; conversely interventions or information based on experience and extrapolation should be viewed with caution (Guyatt et al., 1992). Self-styled as a medical paradigm-shift to scientific clinical medicine, the individual being treated or investigated could now expect to reliably receive the most effective and safest intervention.

The central tenet of EBM was in what it took as the best evidence for clinical decision-making. It was therefore both at once a methodology – how to do medicine – and a philosophical/moral statement about which evidence should be used and which should not. As early critics outlined, of course medicine was always based on evidence (Grahame-Smith, 1995). However, EBM was presented as a step-change in that at its core was increased objectivity delivered by suitably designed studies that minimised bias and confounding factors to represent things as they really are and thereby improve patient outcomes. The EBM Working Group therefore introduced what it saw as not only a new way but a better way, which by implication doctors should be using to deliver maximum patient benefit, thereby introducing an implied ethical/moral imperative.

From an epistemological perspective, the new idea of evidence was designed to set EBM apart from rationalist models of medicine and those predicated on experience, eminence, and accepted opinion (Howick, 2011). Rationalist medicine rested on the idea of evidence that the underlying mechanisms of disease must be understood before an intervention can be said to have worked – that is, only by knowing what has gone wrong (pathology) can the remedy be administered and subsequently be said to have worked. Experiential medicine worked on an apprenticeship model where only through direct and repeated experience of disease could a doctor master medicine and then go on to impart accrued wisdom to others. Whilst accepting that some doctors were performing a style of EBM, this was in no way consistent across the profession with observed variations in how patient values were integrated into care, and differences in provision of interventions between individual doctors (Sackett et al., 1996; Weatherall, 1994). Expert consensuses were both widespread and much valued (Goodman & Baratz, 1990) and textbooks were found to be based more on opinion than clinical evidence (Antman et al., 1992). This led to EBM proponents characterising medicine at the end of the 20^th^ century as overly reliant on both mechanistic reasoning and expert opinion or consensus (Chalmers, 2002; Sackett, 2008; Smith & Rennie, 2014).

### EBM in its infancy – Mark I

Greenhalgh & Worral (1997) have documented the evolution of EBM from its inception to contemporary practice and have distinguished three separate, but interlinked approaches: Mark I, II, and III (Greenhalgh & Worrall, 1997). Evidence-based medicine Mark I ran from the early to mid 1990s, Mark II from the mid to late 1990s before Mark III appeared at the turn of the century. They characterise Mark I EBM as:

The good doctor is one who can *access* and *appraise* the evidence using a daunting armamentarium of statistical tricks such as risk ratios and confidence intervals. The bad doctor, by implication, is one who doesn’t know his sensitivity from his specificity and thinks that Bayes is the stuff they cover snooker tables with.

Sackett and others, following on from the 1992 article, toured hospitals in North America and Britain with their evidence-based trolley, attending to patients on wards and demonstrating the value and applicability of EBM in the clinical setting (Ellis et al., 1995). It was generally enthusiastically adopted by both senior and junior professionals who welcomed its democratising effects and non-dogmatic approach (Smith & Rennie, 2014). This conception of EBM brought a flurry of studies expounding its success, showing that a high percentage of medicine could be regarded as evidence-based (Gill et al., 1996; Geddes et al., 1996; Ellis et al., 1995).

However, as to be expected, there were stringent, sustained and at times withering criticisms aimed at this new medicine. Clinicians, especially more experienced ones, saw EBM as implying that medicine before EBM had been an unscientific, anecdotal mess of paternalistic eminence-based medicine (Howick, 2011). Accusations of arrogance, high-handedness, disrespect for experienced clinicians, impracticality, hubris, obsession with randomised trials over patient outcomes, and cookbook medicine abounded in letters to journals and, notably, in a *Lancet* editorial (Smith & Rennie, 2014; Lancet, 1995; Broom et al., 2009; Howick, 2011; Hamilton, 2002). The criterion for evidence-based was challenged as being too lax, with inclusion permitted for one randomised controlled trial (RCT) showing a pharmacological agent was superior to placebo, even if this was not repeated in subsequent studies (Greenhalgh, 1996).

Sackett and colleagues attempted to rebuff these criticisms largely on the grounds that they were based on a misunderstanding of the discipline and, to a certain extent, a resistance to change and perceived criticism of previous practice (Sackett et al., 1996). EBM did not explicitly say that clinicians were not using evidence, but rather that they lacked the frameworks and skills to apply it in a systematic and consistent way (Liberati & Vineis, 2004). Nevertheless, these were things not easily adopted by experienced clinicians and something needed to give to preserve the good of EBM but make it palatable to the profession more widely. The most enduring criticism was that this objectivity-centric medicine de-valued clinical expertise and reduced individual patients to objective parameters, ignoring their interest in their own medical care (Sackett et al., 1996; Hasnain-Wynia, 2006; Peile, 2013).

### Evolution of EBM – Mark II

In the mid-1990s, proponents of EBM updated its definition to include patient values and preferences alongside objective evidence and clinical expertise. The revision of EBM was laid out in Sackett et al. (1996) in the paper ‘Evidence-based medicine: what it is, and what it isn’t’:

Evidence based medicine is the conscientious, explicit, and judicious use of current best evidence in making decisions about the care of individual patients. The practice of evidence-based medicine means integrating individual clinical expertise with the best available external clinical evidence from systematic research. By individual clinical expertise we mean the proficiency and judgment that individual clinicians acquire through clinical experience and clinical practice. Increased expertise is reflected in many ways, but especially in more effective and efficient diagnosis and in the more thoughtful identification and compassionate use of individual patients’ predicaments, rights, and preferences in making clinical decisions about their care.

Greenhalgh & Worral (1997) describe this second iteration of EBM as follows:

Mark II: The appraisal and application of scientific evidence are not to be pursued willy-nilly … but should be conscientious, judicious and explicit … and give considerable ground to the art of medicine.

However, this brought its own issues. In conceding to clinical experience and patient values, EBM became somewhat less distinct from other ways of doing medicine by:

… allowing the clinician at the bedside have his cake and eat it. If the evidence fits with your old-fashioned and idiosyncratic way of practising, then go ahead and use it. If it does not, that’s fine too: you are, after all, entitled to use your experience and judgement in place of randomized controlled trial evidence or in contravention of any particular set of guidelines in particular circumstances (Greenhalgh & Worrall, 1997).

Moreover, little detail was provided around how to integrate these divergent aspects of best evidence, clinical expertise, and patient values, especially which should take more prominence in the individual clinical encounter. EBM proponents had written a series of textbooks and articles centred on critical appraisal of primary evidence but failed to define expertise and values, or how, where, and with what weighting they should be integrated, in anywhere as near as much detail. As such, criticisms of leaving doctors to it, or EBM as the ’Emperor’s new clothes’ were difficult to fully defend (Dowie, 1996; Fulford et al., 1996; Greenhalgh & Worrall, 1997; Greenhalgh et al., 2014).

### Contemporary EBM – Mark III

The move towards Mark III began towards of the end of the 1990s and carried over into the new millenium.  It has been described as ushering in a contemporary version of EBM, namely:

Mark III: Retains the respect for high-quality epidemiological and clinical trial research of the Mark I model. It retains the criteria of flexibility and patient-centredness introduced in the Mark II model. But it introduces an important additional notion – that the clinician’s entire decision-making sequence can be subjected to full scientific scrutiny (Greenhalgh & Worrall, 1997).

This has been described as a unifying paradigm, combining the features of strong epidemiological research-based evidence of Mark I (Culyer, 1992) with clinical expertise and clinical- and cost-effectiveness of Mark II (Dowie, 1996; Ham, 1995; Fulford et al., 1996; Hope, 1995). Decision-making by clinicians – that is, clinical expertise – is now an important area of research to identify factors in, and their effect on, clinical decision-making. Through this research the question as to how truly evidence-based clinical medicine is or, more importantly, can be expected to be, may be answered (Thornton et al., 1992; Greenhalgh & Worrall, 1997). The successful integration of clinical expertise can be judged by outcomes delivered to individual patients, thereby completing the EBM circle.

### EBM and Evidence-based Healthcare (EBHC)

As EBM has progressed, so has its reach into other aspects of healthcare. EBM as narrowly construed (Mark I) has been described as, to an extent, occurring before the clinical encounter (Thomas, 2016). EBM is the process of clinical decision-making that prioritises more reliable, objective evidence in favour of less systematically-synthesised, less reliable evidence – the founding idea of clinical epidemiology. In this methodology of accessing and appraising evidence, the individual patient is not yet a feature. Rather, individual patient circumstances and values, along with clinician expertise, are integrated with EBM to create evidence-based healthcare (EBHC), where individual patients undergo interventions. Therefore, whilst criticisms can be aimed at EBM these will be of a more scientific-method nature, whilst those of EBHC will be more related to practical and applicatory aspects but the two should not be confused.

Whilst there may be a case to be made for an EBM/EBHC divestment, we contend that such a differentiation is somewhat futile for the simple reason that without EBM, EBHC does not exist; the whole pillar of EBHC is dependent on EBM as its defining feature. EBM has always been about practical medicine – what works for patients – and simply because a central aspect of this involves systematic referencing of research literature it is fallacious to think this happens in some abstract sense before a clinical encounter with an individual patient. Divesting EBM from EBHC does not in some way protect EBM or EBHC from critique – it is just that the nature of the critiques will be different. Interrogation of EBM/EBHC’s different strands pose important questions about EBM/EBHC’s ethical aspects. Given our view that EBM and EBHC are used interchangeably and a significant difference between the two cannot feasibly be said to exist, we will continue to use EBM in the proceeding discussions.

### Uptake and practical progress of EBM

Whilst as a movement EBM has seen significant changes to its definition since inception, there is no doubting its influence in the human medical world in the past three decades. It now pervades almost all levels of contemporary healthcare. Doctors are given guidelines, protocols, and best practice outlines to assist in applying EBM (Hasnain-Wynia, 2006). With the exponentially rising cost of healthcare provision, healthcare and commissioning institutions use EBM to assess new interventions, formulate guidelines, and decide on funding allocation (Hasnain-Wynia, 2006; Muir Gray, 2004). Bastions of EBM such as the Cochrane Collaboration publish regular updates on the benefits of interventions (Gartlehner & Flamm, 2013). Clinical researchers have adopted more standardised protocols for assessing efficacy of treatments and diagnostics, study design, statistical analyses, and publication of both positive and negative trial results (for example, CONSORT and STROBE guidelines). Many educational institutions have adopted problem-based, critical appraisal-centred curricula, moving away from a paternalistic eminence-based method, for teaching undergraduate medicine courses (Petersen, 1999). The methods and model of EBM have leaped outside of medicine into social science, political policy-making, and even chaplaincy (Sutcliffe & Court, 2005; O’Connor, 2002). EBM comfortably represents the most significant shift in clinical medicine in the last half-century (Broom et al., 2009; Zimerman, 2013; Greenhalgh et al., 2014).

## A brief history of evidence-based veterinary medicine

Interest in applying EBM to the veterinary sphere first emerged in the late 1990s partly influenced by the increasing popularity of alternative veterinary therapies (Milstein, 2000; Roen, 2001; Fogle, 1998; Malynicz, 1998; Roper, 1998; Jones, 2000). There was a robust ethical argument that only interventions based on strong evidence should be used and that society would both expect and demand that veterinary clinical decisions were based on science if Aesculapian authority was to be maintained (Shaw, 2001; Ramey & Rollin, 2001). Researchers began to look afresh at the accepted clinical doctrines and like in EBM uncovered swathes of leading articles, books, consensus statements, and guidelines that were based on nothing more than anecdote and opinion (Olivry et al., 2001).

The discipline was formalised in 2003 with the publication of *Handbook of Evidence-Based Veterinary Medicine* where a simple definition of EBVM was detailed – using the current best evidence when making clinical decisions – whilst omitting the cornerstones of clinical expertise and patient values (Holmes & Cockcroft, 2004; Cockcroft & Holmes, 2003). It was therefore similar to EBM Mark I.

EBVM gained significant momentum in the following years. Evidence-based reviews of common conditions began to appear and journals began to request papers that met EBVM guidelines (Rossdale, 2003a; Rossdale et al., 2003b; Olivry & Mueller, 2003; Mueller, 2004). Papers outlining how to apply EBVM were published, including literature searching, critical analysis, study design, and statistical methods (Faunt et al., 2007; Murphy, 2007; Holmes, 2007; Trevejo, 2007; Evans & O’Connor, 2007; O’Connor & Evans, 2007; Cockcroft, 2007; Robertson, 2007). Systematic reviews in areas of veterinary medicine and surgery were published (Olivry & Mueller, 2003 Fahie & Shettko, 2007; Lunsford & Mackin, 2007; Aragon & Budsberg, 2005; Dru Forrester & Roudebush, 2007) and 255 had been produced by 2012 (Jorge & Pfeiffer, 2012). Conferences specific to EBVM were convened in the USA, leading to the formation of the Evidence Based Veterinary Medicine Association (Vandeweerd et al., 2012b). The Centre for Evidence-based Veterinary Medicine (CEVM), the first of its kind, was opened at the University of Nottingham in 2009, and has been instrumental in the production and dissemination of evidence summaries aimed at general practitioner veterinarians in the form of Best Evidence Topics (BETs) which have been used in emergency human medicine for several years. Since 2017, the RCVS has financed an independent charitable arm, RCVS Knowledge, as a global intermediary for EBVM, including a repository of Knowledge Summaries and publishing the first dedicated veterinary journal for EBVM, *Veterinary Evidence*.

The importance of EBVM in the UK veterinary profession was formalised in a 2012 position statement from the RCVS:

The RCVS expects veterinary surgeons and veterinary nurses to make clinical decisions according to their professional judgement, based on the best available evidence at the time … in order to be considered fit-to-practice (*sic*), veterinary practitioners hold the responsibility to ground their decisions on sound, objective and up-to-date evidence, when available. The safeguard of the welfare of animals … is indeed dependent on it (Jorge and Pfeiffer, 2012).

In this statement, EBVM is, in broad brushstrokes, aligned to the EBM method by inclusion of “best evidence” and “professional judgement” in clinical decision-making. Moreover, it appears to go further in explicitly tying the use of EBVM to clinical competence and fitness to practise, something that does not apply to human doctors (General Medical Council, 2013). In doing so, the RCVS introduced an ethical imperative to use EBVM as it is regarded as better for veterinary patients.

With this momentum came improved definitions of EBVM, thereby avoiding the self-critical cycle seen in human medicine and rapid adoption of a form more akin to Mark II EBM. From the CEVM:

Evidence-based veterinary medicine is the use of best relevant evidence in conjunction with clinical expertise to make the best possible decision about a veterinary patient. The circumstances of each patient, and the circumstances and values of the owner/carer, must also be considered when making an evidence-based decision (Centre for Evidence Based Veterinary Medicine, 2015).

Later, protagonists launched the evidence-based veterinary medicine manifesto in 2020 with a stated aim to “use the best evidence to treat and care for animals”; in somewhat of an echo of early EBM, the manifesto was designed to move on from a philosophical debate of what EBVM is – or is not – and help the profession with “getting on and doing something about it” (Veterinary Record, 2020).

EBVM has yet to move to embracing the assessment of clinical decision-making within a veterinary intervention (EBM Mark III). We believe that this may be due a combination of EBVM’s relative infancy, the smaller size of the profession compared to human medicine, the structure of the profession in terms of many small clinics, insufficient interest in this area of research and difficulties in systematically tracking short- and long-term outcomes in clinical care. With increasing corporatisation of the profession leading to large groups of clinics linked electronically and the emergence of epidemiological big data initiatives such as VetCompass who have produced several publications in the past few years, this may be expected to change in the future.

Despite its apparent official adoption and acceptance, EBVM has attracted criticisms from within the profession, though less voluminous than in the human sphere (Mills, 2015; Schmidt, 2007). The relative incommensurability of EBVM with EBM has been highlighted. Whilst the professions share some similarities, they also possess a fundamental difference in terms of species treated: most (but by no means not all) human patients can self-report symptoms and values. Therefore, the clinical expertise required is different in nature and there are issues that come with reliance on a proxy (owner) for clinical history reporting of symptoms, limitations on information from clinical examination and the non-verbal status of all veterinary patients. The lack of a good quality evidence base, lack of epidemiological analytical skills, accessibility of primary research, the laborious nature of EBVM methodology for first opinion veterinarians and the production of most research not representative of more than 95% of the veterinary workforce (and by extension patients) have also been highlighted as practical barriers to its whole-scale adoption (Vandeweerd et al., 2012a; Vandeweerd et al., 2012b; Vandeweerd et al., 2012c; Mills, 2015; Nielsen et al., 2015). Some of these aspects will be explored in more detail later.

## The philosophy of evidence in EBVM

In discussion with key stakeholders including veterinary anaesthesiologists, orthopaedic surgeons, nurses, and hospital leadership, countermeasures were proposed based on the results of the root cause analysis. Utilising an impact effort matrix, the first intervention that was trialed was improving the current communication protocol. For each TPLO case, prior to induction anaesthesia service personnel would send a notification through the intrahospital messaging system stating that the patient was ready to be induced. This would allow orthopaedic service and operating room personnel to be present and prepared before anaesthesia time officially began. This would decrease nonoperative anaesthesia time that was previously used to organise personnel. This described communication protocol was developed in December 2021 and TPLO cases were reaudited following this intervention from January 2022 through April 2022. A potential barrier for this new communication protocol would be a lack of participation. There are many groups involved in making this protocol successful so if participation was lacking from any of these groups, we would not see the overall change in nonoperative anaesthesia time.

### Objectivity and truth in EBVM

Taking clinical evidence in EBVM, the overarching epistemological claim is that the more objectively the evidence is synthesised, the more objective and therefore reliable the results; these can then be taken as stronger evidence for, say, an intervention. We will not examine the philosophy of truth in this paper, for it is not necessary to contend that objectivity is linked to truth in EBM/EBVM (Wieringa et al., 2018), only that the closer to the truth evidence can be, the greater justificatory power it confers to clinical knowledge. EBVM via its triumphing of more objective clinical research regards evidence from such research as truer than that from less objective sources such as experience and anecdote.

It is useful to examine what measures are taken to improve objectivity in production of clinical evidence and challenges to these concepts. Taking the RCT – generally regarded as most objective method of  evidence synthesis – as an example, controls are applied in study design and analysis. Randomisation attempts to control for the confounding factors of known and unknown variables by (approximately) equally distributing these between groups to prevent selection bias; blinding looks to prevent the subjective distortion of analysis of outcomes by investigators. The objectivity of these measures has been challenged. The act of randomisation may not evenly distribute confounding factors as they can only be probabilistically evenly distributed (Worrall, 2002). The RCT is also a point-in-time estimate; it is not performed indefinitely, which would allow it to gain some external validity as to the even distribution of confounders (Worrall, 2009). To manifest internal validity, RCTs must restrict input (randomisation, homogeneity of population) and this can mean that the external validity or generalisability of the results is limited – “they will at best *vouch* for the conclusion rather than *clinch* it” (Cartwright, 2007).

Post hoc statistical analysis attempts to further control for confounders. Classically, the p*-*value for statistical significance is set at 5% (P = 0.05), which is to say that if P < 0.05 then the differences observed in the data are deemed to be statistically significant, and the null hypothesis rejected. The origins and usefulness of the p-value have been questioned with the 5% cut-off variously described as arbitrary, due to folklore, plucked from the air, and too easy to find significance at (Greco, 2011; Rosnow & Rosenthal, 1989). A value judgement has ultimately been made to set it at 5% and statistical significance therefore has a subjective foundation. The complementary nature of power to the p*-*value means that despite a good quality study being performed, insufficient power (too few participants; too large an effect expected) can lead to missing significance cut-off. The epistemic significance that can be applied to the p*-*value has also been much debated, with opinions split as to whether it should apply strictly (null hypothesis rejection) or epistemically (indicates the intervention studied is efficacious) (Mayo & Spanos, 2006; Spanos, 2013; Wasserstein, 2019; Verploegh et al., 2022).

Evidence-based Veterinary Medicine aligns its view of evidence to elements of objectivism, the school of thought that maintains if phenomena are reported stripped of bias it allows an observer to see features of the world as it really is. In assuming the view from nowhere faithfulness to facts is assured by absence of commitments (theory-ladenness) and values, and freedom from personal biases. It has been described as both the basis of and ultimate aim of the scientific method (Longino, 1990).

In the philosophy of science this view has largely been dismantled by identification of subjective processes at the heart of scientific methods and reasoning, much as highlighted in the RCT discussion above (Goldman, 1993; Goldenberg, 2005; Reiss & Sprenger, 2018). Scientific observations are theory-laden by background knowledge, perceptions, and assumptions, meaning that no data from them, regardless of the nature of generation, or careful experimental controls applied, can ever give objective unmitigated descriptions of the objective nature of things. The view from nowhere of objective facts about the world is only possible in the absence of an observer, or that all observers are connected by a universality of experience. Clinical research is directed by theory and the subject of research determines methods employed which ultimately influences the evidence produced (Kuhn, 1996). Science has been described as socially or culturally influenced (Longino, 1990; Goldenberg, 2006). Theories are underdetermined by the data, so no theory can stand or fall by the evidence alone as ordinarily more than one theory can plausibly explain the data observed (Duhem, 1982; Quine, 1960).

More recently, and specifically to the practise of EBVM, the idea that once we can defeat all sources of bias, the truth will uncomplicatedly be revealed and subsequently lead to better decision-making has been heavily criticised (Wieringa, 2018). In part, this is because truth is not a simple concept but is subject to cultural, metaphysical, and contextual factors that can alter its meaning according to circumstance. Bias has been similarly characterised as heavily affecting a method of questioning (research) so on a macro level the attempts at eliminating bias in pursuit of objectivity is inherently biased in and of itself, based as it is on belief rather than any degree of compelling evidence (McKenzie, 2014).

Proponents of EBM/EBVM have denied that it aims to be objective, only relatively so (Sackett 1996; Worral, 2007; Bluhm, 2011). It is obvious that even on a superficial reading, the EBM/EBVM stance on evidence is relative and always has been: evidence can have a greater or lesser objectivity, and therein have a greater or lesser quality and justifiable applicability to the clinical case. It is desirable for evidence to be as objective as possible only insofar as to ensure that the effect seen in a trial or study is due to what is being studied rather than confounding factors. Criticisms of Mark II EBM, namely that it permitted flexibility as to when apply the evidence or not according to circumstance, are difficult to defend against; however, ultimately such plasticity comes at a cost of robustness. If objectivity in EBM/EBVM is grounded in the processes of study design and statistical control, and these can be determined as somewhat subjective then quite how objective (or how more objective) EBM/EBVM is can be questioned. The epistemology of EBM/EBVM does not define a new truth or objectivity, but rather places value judgements on objectivity, much akin to the way that the natural sciences do.

### Inductive reasoning in appraising and applying evidence – increasing the risk of bias

Evidence-based veterinary medicine does not only take a position on the idea of evidence but also describes how evidence should be used in the triumvirate of evidence, veterinarian expertise, and patient circumstances. To achieve this, EBVM veterinarians carry out some heavy practical epistemological lifting, using techniques that are not inherently objective, namely induction and inference to the best explanation (IBE). As evidence does not entail a best approach then deductive reasoning (where the conclusion is entailed in the premises) is rarely if ever utilised (Djulbegovic et al., 2009; Shahar, 1997; Popper, 1968; Little et al., 2012); whilst it seems somewhat fanciful that the originators of EBM/EBVM envisaged a deductive reasoning method of clinical medicine, nevertheless objective evidence would be expected to entail a conclusion more than less objective evidence.

Induction or IBE is required when appraising even the more objective (as defined by the EBM/EBVM theory) studies. Induction, understood as causal-inductive processing of evidence, details reasoning from a few observed cases to the general case (Achinstein, 2005); it has been described as the primary method of reasoning in science and medicine (Djulbegovic et al., 2009). Such would be seen, for instance, in application of study results to patients not involved in a clinical study.

As described by Lipton and others, IBE has close similarities to induction (Lipton, 2000; Harman, 1965). It differs in that it places evidence within the context of what is already (putatively) known in order to explain, in medicine, the clinical manifestation of disease, therapy effects, and so on. In EBM/EBVM, IBE is employed when selecting the best or most likely-to-be-true hypothesis for the signs or effects seen. More controversially, it may also be employed in the process of meta-analyses or systematic reviews (Silva & Wyer, 2009).

We will now expand on how when a clinician is required to apply the evidence, the reasoning becomes almost exclusively inductive or inferential; further, there remains a paucity of guidance on how this can be performed without significant bias, the very feature that EBM/EBVM aims to minimise.

Inductive reasoning and IBE are important because they pervade EBM/EBVM both in appraising and applying study findings to individual patients. As we have described, the higher objective claims of EBM/EBVM involve a significant amount of inductive reasoning. The crux is that both these forms of reasoning cannot be justified rationally or logically. Inductively reasoning that your computer will not blow up when you turn it on today because it has not exploded each day for the last few years when turned on does not guarantee your belief it will not blow up today. The evidence you have is indicative, not proof. The epistemological debate around induction and IBE has  played out over many years and continues to do so (Lipton, 2000); its details are not required here. Rather, it is important to recognise that EBVM uses this form of reasoning readily when appraising and applying evidence to clinical cases and this introduces limits to what can be achieved – at best the outcome can be probabilified not ensured. On this level, it should not dismay advocates of EBVM too much as the logical end point of claiming to guarantee an outcome is akin to being able to precisely predict the future in every case – anyone claiming such would normally expect to be met with severe scepticism at the very least.

Inductive reasoning and IBE are more problematic in their tendency to introduce a plethora of cognitive biases in the decision-making process. McKenzie (2014) details ten such possible biases in veterinary medicine, drawing on decades of research in the human field (Kahneman, 2011; Croskerry, 2002). In our experience, of particular prevalence in veterinary medicine is confirmation bias, where evidence is sought to confirm a hypothesis rather than disprove it, such as assuming when a patient improves we were correct in our diagnosis and/or therapy, where the patient may have improved regardless of intervention. Such a bias highlights the pitfalls of induction and IBE. Biases are important as they increase the chance of errors (Croskerry, 2003).

Biases in reasoning both about evidence and how it is applied are somewhat built-in to human psychology (Kahnemann, 2011) and so are not an issue inherent or exclusive to EBVM. But EBVM does not have, as yet, any new practical approaches to minimising them other than in the synthesis of evidence which, as discussed, requires some degree of induction and so is not as objective as it first may appear even for study types such as RCTs. The process of integrating clinical expertise and the circumstances and values of the animal owner have been only briefly described both in EBVM (and EBM).

McKnzie (2014) describes one possible solution for EBVM based on dual process theory of decision-making which has become the dominant model in cognitive psychology over the last few years. Briefly, it divides decision-making into system 1 and system 2. System 1 is rapid, unconscious, intuitive, heuristic and subconscious – in veterinary medical practice it allows a quick diagnosis via a variety of pattern recognition, experience, and avoidance of an explicit reasoning process. System 2 is a conscious, effortful, systematic process by which learned techniques are used to actively reason to, say, a diagnosis – in current veterinary medicine this would be by construction of problem lists, differential diagnoses, and reasoning to exclude or include possibilities. Using system 2 may, in some circumstances, reduce bias-based errors (Croskerry, 2003). However, the sheer time it takes means it is a poor fit for first opinion practice, where most presentations are mild, information is limited and a diagnosis is not always required for an appropriate intervention (May, 2015, McKenzie, 2014).

In EBM, the use of clinical guidelines which summate the evidence into usable documents such as decision-trees or algorithms for conditions are commonplace (Greenhalgh et al. 2014). In essence this subcontracts system 2 thinking to the creators of the document, meaning clinicians can efficiently and effectively access the best evidence for a clinical case. Such evidence summaries are moving apace in EBVM also.

The proposed system 2 solution only deals with clinical expertise; we are not aware of any scheme describing how to integrate owner circumstances and values into a clinical decision-making. The influence of owners on decision-making is significant. Non-human animals lacking autonomy to make decisions about their own care represents a fundamental difference between EBM and EBVM; although there are numerous scenarios in in the former where patients lack the capacity to make decisions about their own care, in EBVM we argue all patients lack such capacity. Owner influence has been described by Yeates & Main (2010) as falling into categories of beliefs, values, financial situation and logistical circumstances which whilst important are not strictly veterinary medical considerations. Whilst not commenting on EBVM, Rollin (2006) has described the veterinarian’s role in integrating owner circumstances as varying from a garage mechanic fixing a problem to a paediatrician advocating for the animal, sometimes within the same individual case. How to practically perform these roles has not been well described.

We appreciate that much of the preceding commentary deals with practicalities of application, but they are relevant to the philosophy of EBVM in that they exemplify a high degree of subjectivity and potential bias within its methodology. As a result, we contend that the central claim of EBVM – a more reliable, better way of doing veterinary medicine – rests ultimately and almost exclusively on the use of results from more reliable studies such as RCTs in preference to other evidence. It does not appear, at this stage, to have anything revolutionary to improve decision-making or integrating owner values and circumstances, which are somewhat left to inductive reasoning and inference. These areas have been described as constituting the majority of the clinical encounter and intervention in EBM (Worral, 2002; Gupta, 2003) and we contend the same is true in EBVM. Once we examine, even briefly, the objectivity of evidence theory and its relation to truth, the central tenet is of greater objectivity is not as compelling as it immediately sounds. It has been generally accepted that the original proclamations of EBM as a paradigm-shift in medicine were over-ambitious (Greenhalgh et al. 2014); we therefore urge EBVM, learning from the history of EBM, to not be promoted in a similar way.

Much of the above criticism around reliance on inductive reasoning and inference is not exclusive to EBVM, pervading as it does most decision-making across the both the sciences and the arts. However, we disagree that this makes it a “parasitic attack”, as has been described by EBM commentators (Thomas, 2016), for a very simple reason. The subjectivity, bias-heavy, illogicity issues with inductive and inferential reasoning in the philosophy of science are extensively documented (Popper, 1968; Kuhn, 1996). They are used extensively in EBVM, a movement with a major claim of being more reliable and objective but without, currently, a new approach to reasoning that justifies such claims.

However, as seen in EBM Mark III, the success of clinicians’ reasoning can be studied and differences in success can possibly be explained. It is unlikely that induction or IBE can be shown to be reliable all the time or even be superior to other forms of reasoning (whatever they may be) but it may be possible to show that they achieve a repeatable probability of succeeding. It may be an unintended but welcome consequence that a movement yoked so closely to objectivity ultimately assists in improving methods of non-objective reasoning.

### The limits of evidence in EBVM

When assessing the epistemological value of Information generated by EBVM, the question of what the evidence is for is often posited: the real-world value of evidence in in terms of what it refers to and how it can be used. Returning to the RCT, it may in part depend on our reading of the results, whether on a strict interpretation (accept/reject the null hypothesis) or an epistemic reading of being able to reject the null hypothesis and via induction or IBE estimate what may underlie the effects seen (or not seen) – such is the purview of discussion sections in most papers. Regardless of which is used, there are still epistemic limits of evidence that can be appreciated by considering the difference between efficacy – results demonstrated in clinical research – and effectiveness – how the intervention works in the untested patient in the real world (Ashcroft, 2002).

In almost all cases, the individual patient subject to an intervention is an untested case and therefore the judgement of its effectiveness rests on inductive reasoning – it worked on others so it should work on the next patient. In practice, it is seeing that something works, confirming the validity of the inferential step from research efficacy to real-world effectiveness, providing post hoc empirical confirmation of success. Applying the intervention will only probabilistically cause an improvement in the condition being treated as the evidence cannot ensure success.

Consider an intervention for a cat with disease X. The evidence from multiple RCTs gives a 92% chance of full recovery with treatment Y. Pretreatment it is possible to state that the treatment is efficacious in 92% of patients but we cannot say that it will be effective in this individual cat. We cannot give the cat the treatment 100 times in parallel, where the cat would recover 92 times and not recover 8 times. Even assuming the cat is biologically similar to those in the RCTs, we still cannot predict whether in this single case it will recover from X with treatment Y.

Effectiveness can therefore only be ascertained after the intervention has been carried out. For an individual clinician, or the epistemic community, the response of the individual patient(s) will inform a notion of clinical effectiveness; experience strengthens or weakens the evidence for induction to future cases. Equally, however, regardless of the amount of experience, it is not possible to generalise with certainty to future untested cases, since this takes us back to the fantastical realms of fortune telling.

Evidence of any source therefore will always have an epistemic limit unless perfect future-prediction is assured; whilst more and more detailed evidence can increase the probability of clinical success, it can never guarantee it. It is not the fault of EBVM that we cannot accurately predict the future, but the epistemic limits of evidence are important in two ways. Firstly, how much efficacy can tell us – as the simple example above details it matters for the individual. Secondly, how effectiveness is confirmed or invalidated by clinical experience. So-called truth tracking of effectiveness rests heavily on a source of evidence traditionally considered to be low in the hierarchy of evidence in terms of reliability in EBVM.

A related question is whose evidence is it? This is important in deciding how applicable the evidence is to the individual patient and how great an inductive or inferential step is required. A strict reading of generalisability would maintain that clinical research results can only be directly transposed onto the individual patient if the individual conforms to the study group in all relevant aspects, which is implausible in most cases (Black, 1998). For example, most studies into canine myxomatous mitral valve degeneration are carried out on Cavalier King Charles Cocker Spaniels, given the high prevalence and predictable clinical course of the condition in this breed of dog (Häggström et al., 2008; Boswood et al., 2018). Strict generalisability would maintain the results of any studies are only extrapolatable to other dogs of the same breed, age, and with the same level of clinical condition. Using a less-strict generalisability approach, justified by biological similarity between breeds, species, and relatively close evolutionary ties, may allow results to be extrapolated by introducing an extra inferential step. Taking such a step may be justifiable but with each inductive action we are taken away from objectivity and into the arena of reasoning and extrapolation. We contend that this reduces the epistemic power of the evidence further especially when considered along with the efficacy/effectiveness uncertainty already detailed. However, the alternative – not extrapolating from evidence or not having any evidence available from which to extrapolate – is likely even more fraught with issues. For instance, relying on clinical impression or intuition and reasoning from first principles not only places a much greater epistemological pressure on the veterinarian but also introduces far more inferential steps with increased risk of errors. So, whilst it can be recognised that evidence may not be perfect for every individual we propose it is nevertheless more likely to be more reliable than having no evidence at all.

Nevertheless, extrapolation is not necessarily risk-free. For example, the emerging field of pharmacogenetics is uncovering how different breeds of dogs metabolise drugs at different rates with resultant different proportions of active substance levels and half-lives (Fleischer et al., 2008) Furthermore, some breeds of dogs with deficiencies in certain genes (e.g. MDR-1) may cause increased sensitivty to some agents, leading to unpredictable outcomes. (Geyer & Janko, 2012). However, experience-based or eminence-based medicine are similarly not without risk. Even though it is difficult to ascertain relative risks of different approaches to veterinary medical practice, by their nature experience-based or eminence-based medicine rely on the observed experiences of a single or small group of veterinarians which are likely to be not only unsystematic (introducing bias, as discussed) but also limited in case number. It should also be noted that risks of extrapolation have been identified by primary research methods using methodology deemed reliable in the EBVM model.

## Practical problems with EBVM

### Quality of evidence concerns: whose evidence and evidence for what?

A major issue in the veterinary profession is population skew, with most veterinary study participants being drawn from referral populations. An audit of articles in the *Journal of Small Animal Practice* found 93% of papers published in the year 2018 involved exclusively referral-level populations (Mills, 2021). Referral populations are rarely representative of the general population, having a higher prevalence of pedigree animals, with more serious disease states (May, 2015). They have been characterised as mainly the inexplicably ill which require a definitive diagnosis before targeted therapy, something which is poorly matched to most first-opinion work where symptomatic therapy is often prescribed for animals (May, 2015). Specialist-produced evidence may detail interventions unavailable to the wider profession due to complexity, required veterinary competence, or specialist equipment. If we add this skew to the issues around epistemic limits of evidence, especially around the known and unknown biological differences between breeds and species, then the inferential steps to map studies onto the general population or unseen cases are doing more and more work. The question of whose evidence? should be a concern of most published veterinary evidence especially where more than 95% of UK veterinary surgeons work in first-opinion practice (RCVS, 2019).

Skew in study populations is well-documented in human healthcare. It has been estimated that 90% of studies look at diseases of the wealthiest 10% of the Western population (Goldenberg, 2009); over 90% of RCTs are run on young white males (Dresser, 1992) (Weijer & Crouch, 1999); and fewer than 10% of studies identify race in participants (Weijer & Crouch, 1999). These necessarily map poorly onto the diversity of unseen primary care patients who are often multimorbid, of older age, from ethic minority groups, female, may struggle with appointment availability and medication compliance, and be affected by complex socioeconomic and psychological components of disease (Rogers, 2004).

Such patterns in the human and veterinary medical fields may be explained by different factors. In human medicine, pharmaceutical and surgical companies have a long-established significant influence on the research agenda – bringing new drugs or techniques to market is profitable (Goldacre, 2009). In the veterinary field this also exists, many RCTs looking at medical interventions are funded by pharmaceutical companies and these sponsored studies are more likely to report positive outcomes (Wareham et al., 2017). In surgery, the relatively large number of studies into canine cruciate disease, may be in part explained by the disease’s financial value to the profession – the total cost to owners, as charged by veterinary practices, for the management of cranial cruciate disease in the US during 2003 has been estimated at $1.32 billion (Wilke et al., 2005). A recent study showed that in UK dogs undergoing surgical management of the disease, 62% had more advanced costly procedures (Pegram et al., 2024) even though clear superiority of one technique over another has not been convincingly demonstrated (Mills, 2021). Surgical procedures often make up a significant proportion of income in university and referral centres. This potentially creates a risk or surgical technique bias which may influence the research agenda in turn.

Evidence in EBVM is both low in volume and quality (Cockcroft & Holmes, 2003; Dean, 2017; RCVS Knowledge and Sense about Science, 2019; Mills, 2021). Some authors have described this as evidence often being contradictory, especially when pitching external evidence against personal, undocumented and unsystematic clinician experience (Mills, 2016; Dean, 2017). Veterinarians can in most cases find evidence for any intervention they may wish to pursue. Rather than representing a beacon of clarity, the evidence, and subsequent decision-making, is often fuzzy and outcomes are not as highly probabilified or predictable as in the human sphere. For example, an analysis of the Knowledge Summaries in *Veterinary Evidence* in 2023 shows that out of a total of 19 companion animal summaries, 74% concluded insufficient evidence was available in quality and volume to formulate a recommendation. In 12/19 of these summaries evidence was graded as weak overall, moderate in 5, and no studies were available in 2 summaries. ^[1]^

These findings do not diminish the value of the summaries, but do starkly reflect that there is insufficient volume or quality of evidence, or that appropriate therapy does not yet exist (or has not been adequately elucidated by an appropriate study); regardless, it does reduce their usefulness to veterinarians looking for an indication of how best to manage a certain case.

The relatively dichotomous (first opinion/specialist) make-up of the veterinary profession leads to concerns about to who the evidence in EBVM refers and subsequently what epistemic power it carries (evidence for what?). This is highlighted in the difference between evidence for medical and surgical interventions.

Surgery represents a difficult area in which to apply EBVM as it does not lend itself easily to study by RCTs. As such, the strength of justification for an intervention may be reduced (Stirrat, 2004). Surgical RCTs are problematic as blinding, randomisation and equipoise are difficult to achieve; it may be unethical to continue the trial if one intervention is deemed superior and the use of sham operations as a placebo may be difficult to justify. Surgery also relies heavily on the manual, visual and specific mental skill competence of the individual surgeon; equipment and ancillary care facilities may also be a factor in procedure success (Meakins, 2002; Stirrat, 2004). These practical and ethical issues mean that most surgical evidence is limited to a case series performed by one or a handful of surgeons, limiting, we contend, the epistemic value and transferability of such evidence.

Whilst studies in the field of medicine are still mainly produced by specialists, in contrast to surgery the treatments described are far more transferrable to less well qualified veterinarians – it may be as straightforward as giving a series of injections which is within the purview of all veterinary surgeons. Intrinsically, medical evidence appears to be far more transferrable to the whole profession whilst surgical evidence is far more limited. Ultimately, we believe that pronouncements emanating from surgical studies about best treatment should be tempered with a recognition of the study’s inherent significant limitations both in design and extrapolation.

In EBVM this all plays out against a background of a very different patient population than in human medicine. The epistemic harvest from a clinical encounter between veterinarian and patient is less with reliance on a proxy (owner) for clinical history reporting of symptoms, limitations of the clinical examination, and the non-verbal status of all veterinary patients requiring several inductive steps throughout any veterinary intervention, with less reliable information ultimately available. In human medicine, the same issue exists in a number of scenarios in the unconscious, those lacking mental capacity, children et cetera but in EBVM it exists all the time; whilst not a dichotomy between the professions, EBVM sits at the far end of the spectrum compared to EBM.

Issues discussed in this section we believe raise questions about the relative incommensurability of EBVM with EBM; the differences inherent in both disciplines mean that EBM cannot be directly mapped onto veterinary medicine (Mills, 2015). Whilst some of the issues are around application of the EBVM – especially evidence quality and breadth – some are about fundamental differences between human and veterinary medicine practice. We propose that there is no reason why EBVM cannot be realised, but it should be recognised that with such an array of species and breeds this would be expected to take longer. Studies may need to be better tailored to avoid the difficulties in recruiting veterinary patients, and inherent limitations in the clinical encounter may always limit the epistemic value of history and examination in clinical veterinary medicine which may impact study design and applicability of synthesised evidence.

### Volume of evidence – too much versus too little

In what has been described as both a measure of success and a failing, the volume of evidence available in the human medical field makes the methodology of EBM impractical to the day-to-day care of individual patients (Greenhalgh et al., 2014). As previously described, these are often summarised into clinical guidelines for clinicians to use, however, in an audit of 18 patients in an acute medical ward, 44 diagnoses had 3679 pages of national guidelines appropriate to their immediate care, which would take an estimated 122 hours of reading (Allen & Harkins, 2005). Lengthy reviews that are comprehensive but unusable in practice have been described as being uninformative, dull, and uninfluential (Lavis et al., 2006). There may be a knock-on effect that too much time spent reading guidelines and external evidence limits learning of practical skills essential to becoming a good doctor (Montgomery, 2005; Miles, 2007). Such a problem has been acknowledged in EBM circles, and there has been a push to make evidence more useful, via its presentation in briefer, understandable summaries, diagrams, infographics, and other decision aids (Gigerenzer et al., 2007; Greenhalgh et al., 2014).

In EBVM, the volume of evidence available is far less than its human counterpart (Dean, 2017; RCVS Knowledge and Sense about Science, 2019; Mills, 2021; Cockcroft & Holmes, 2003). However, it is still relatively voluminous for the individual veterinarian to access and appraise the evidence (Mills, 2021) The development of guidelines and evidence summaries (e.g. CEVN BETS, *Veterinary Evidence* Knowledge Summaries), in a similar fashion seen in EBM, aim to reduce the burden on veterinarians to appraise primary evidence.

We are of the strong opinion that the relative lack of good quality veterinary evidence should not result in the simplistic cry for more evidence; focus instead should be on more relevant, easily accessible and usable evidence appropriate for our patients and practice. The unfocussed amassing of evidence, whilst adding to overall knowledge, can in fact lead to more confusion and quickly become an unwieldy mass of studies through which to wade – such is a central lesson of the EBM evolution. This is especially true where methodologies differ between studies, limiting comparative value with established literature or where studies are repeated on an intervention where its efficacy has already been well established – whilst the latter is a desirable thing to do in terms of scientific method, if it comes at the expense of knowledge in under-researched areas it would, from a community perspective, be far less useful.

Recently, changes to evidence synthesis have occurred which may realise the requirement for more targeted evidence using first-opinion practice data. Whilst in its infancy, the nascent movement of targeted trial emulation uses RCT design features to attempt to find causal relationships from epidemiological observational data (Maringe et al., 2020). These have recently been applied in veterinary research (Pegram et al., 2024) and have the potential to avoid the cost, time, recruitment, and logistical difficulties of RCTs inherent in veterinary studies by using data already available from primary veterinary practice. In the human sphere, they have been championed as being more representative of an intervention in the real world, detailing both efficacy and effectiveness more reliably for individual patients outside of a controlled trial (Maringe et al., 2020). In the veterinary sphere, we predict they may be used to counteract the biases of recruitment of a standardised study population (breed, size, age, et cetera) and use of almost exclusively referral populations currently seen in veterinary research, thereby allowing evidence synthesis of greater value to the majority of the profession and the patients they treat.

### How can EBVM individualise care?

Evidence-based medicine has been characterised as lending itself to rule-based medicine, where decision trees, diagnostic pathways, and structured templates can overwhelm local, individualised medicine (Timmermans & Berg, 2003; Glasziou et al., 2013) leading to the loss of the self from medicine (Thomas, 2016). The following of algorithmic rules, whilst providing a safety net of sorts, may stifle clinician development, ability to deal with uncertainty, make nuanced ethical decisions, and individualise care (De Vries & Lemmens, 2006; Glasziou et al., 2013). However, such criticisms are counteracted by others who see them as a misrepresentation of EBM: “Real evidence based medicine is not bound by rules … has the care of individual patients as its top priority … builds on a strong interpersonal relationship between patient and clinician” (Greenhalgh et al., 2014). EBM explicitly cautions against becoming “tyrannised by evidence” (Sackett et al., 1996): it includes the words judicious and conscientious in its definition and the importance of clinical expertise and individual patient views in care have been recognised (Greenhalgh et al., 2014).

Even though the concept of individualism has been recognised in animal welfare science for some years (Yeates, 2013), in EBVM what the veterinary patient may conceivably want from an intervention has received little attention and most definitions of EBVM do not include the concept of patient values or interests, with “circumstances” the closest thing to considering individuality of care (Centre for Evidence Based Veterinary Medicine, 2015). Non-human animals are obviously individual – compare the unowned/feral versus owned cat for instance – and as such it is reasonable to ascribe different preferences to them according to their individual characters. We question, therefore, given that non-human animals are individuals rather than a uniform group of patients, where the safeguards exist within EBVM for individualising care.

Veterinary patients undergoing an intervention are in an unenviable position, being the subject of surrogate decision-making by the veterinarian and (normally) the owner. Non-human animals possess insufficient autonomy, even on the most inclusive of definitions, to make decisions about their own care: they would not present themselves for intervention and are unable to rationalise interventions, vocalise preferences, make cost/benefit decisions, or stop their own treatment (Yeates, 2015). They cannot speak about their symptoms, feelings, preferences, or desired outcomes (Rollin, 2006; Mills, 2015). Decisions are made exclusively on their behalf. In EBVM, as discussed, the veterinarian may assume a number of roles, from garage mechanic who attempts to fix a biological machine to a paternalistic paediatrician acting in the patient’s best interests even when these clash with owner preferences (Rollin, 2006). External non-clinical factors also feed into decision-making, including the competence and knowledge of the veterinary surgeon, and owner considerations such as personal beliefs, financial position, and logistical arrangements (Yeates & Main, 2010). Factors outside individual care we contend may often exert a disproportionately large influence on the decision-making process over and above what may be considered the best intervention for the veterinary patient.  

Given the relative powerlessness of veterinary patients in the course of an intervention, we argue that the requirement for consideration of individual patient preferences in veterinary medicine is at least equal to if not higher than for human patients. Inherent in veterinary interventions is a degree of necessary suffering – presence in the surgery, examination, diagnostic testing, hospitalisation, and treatment can all potentially cause poor mental and/or physical welfare. The mental lives of most companion animals, as far as we understand, are immediate –  a lack of rational thought means current harms cannot be balanced against potential future health gains (Mendl & Paul, 2004; Paul et al., 2005; Mendl et al., 2009); therefore veterinarians are starting from a position of negative welfare equity which increases the moral pressure to improve an individual’s welfare. Veterinary patients’ individuality – regardless of any existing evidence indicating which intervention should be pursued – we argue should be a significant consideration in what is the best approach for the individual patient, therein bringing the patient’s interests under the EBVM umbrella.In the future, we intend to explore how individual interests, external evidence, clinical expertise and owner circumstances can be routinely incorporated into clinical decision-making.

### Diagnosis and treatment

Treatments lend themselves well to efficacy studies, especially prospective RCTs which, to a greater or lesser extent, can remove bias and confounding factors to allow a judgement to be made on a treatment’s efficacy versus the current accepted best treatment or no treatment (Sackett et al., 1985; Vandeweerd et al., 2012a). However, diagnosis is a different process, described as “the art of making decisions without adequate information” (Sox, 1996) which necessitates use of diagnostic art (Lambert, 2006). This is difficult to standardise, and experiential factors have a huge influence on accurate and timely diagnoses (Greenhalgh, 1999).

Nevertheless, EBM/EBVM can be applied successfully to the process of diagnosis by providing information that can help justify a diagnosis and improve accuracy. (Zakowski et al., 2004; Hawkins, 2005). Information about prevalence, disease features and test accuracies from EBM/EBVM studies can be used in Bayesian decision trees (Cockcroft & Holmes, 2003). Information helps ensure that important features are not overlooked (Gawande, 2011). Such information helps with recognition that whilst disease states are unique to the patient, features are shared between patients and the commonality certain aspects can be articulated. Determination of test accuracy, and therefore how much confidence the clinician can have in a result, is an important consideration in the diagnostic process (Zakowski et al., 2004).

Further, application of EBM/EBVM methods to characterise the art of a good diagnostician is potentially illuminating, as discussed previously. Decision-making around diagnosis has been studied, and follows a model of rational optimising, based on cognitive rational models, including decision trees and tables, algorithms and Bayesian probability theories (Eddy, 1990; Abrahantes et al., 2007; Clarke & Rosenman, 1991; Cantor, 1995). Further characterising these, and especially how they develop in clinicians, would potentially mean that teaching the skills required to become better diagnosticians would be possible.

### Poor fit for multimorbidity

In human medicine, as the population ages, chronic degenerative diseases become more prevalent, meaning it is rare to see patients presenting with a single condition that maps neatly onto an evidence-based guideline (Greenhalgh et al., 2014). Multimorbidity affects people differently, and is resistant to application of objective scores, metrics, interventions, or guidelines (Huntley et al., 2012). Management of one disease may worsen symptoms of another, especially where polypharmacy is involved (Duerden et al., 2013).

There have been no direct responses to this issue, other than that human beings are complex biological entities who can be affected by a huge number of diseases, so applying guidelines or studies neatly to each unique individual is unrealistic (Huntley et al., 2012; Greenhalgh et al., 2014). Moreover, it raises the importance of patient involvement in a dynamic decision-making process, and prioritising what matters to them.

Similar considerations are relevant to veterinary medicine, but at present the promotion of the animal’s best interests, what it may prefer or desire, has not been fully explored. For example – would a veterinary patient rather live a shorter life in less pain (through medication) or a longer one in a degree of pain (without medication)? Also, the lack of self-reported information available from animals means that multimorbidity is likely under-recognised. The practice of EBVM is in its infancy compared to EBM and so mapping results onto multimorbid patients is likely some time away until individual studies are performed. However, EBM’s issues in this area serve to the application of studies of a single facet of one disease to these potentially multimorbid patients can open up more complexity even before different breeds, species, owner, and patient circumstances are considered in EBVM. Advances in research methods, such as the target trial emulations previously discussed, may go some way to countering the issue of multimorbidity as long as the temptation to over-control for confounders is resisted – seeing how an intervention performs in a diverse group of veterinary patients will, we argue, produce far more relevant evidence than the relatively sanitised uniform patient populations used in traditional RCTs.

## Ethical features of EBVM – should we use it?

The practice of EBM and EBVM are value-laden in that protagonists describe them   a better way of performing medicine and veterinary medicine. Here we present the ethical case for EBM, which logically also applies to EBVM; an ethical case for EBVM has not been definitively set out in the literature.

The ethical case for EBM is predicated on an assumed epistemological superiority; a simple logic for EBM has been constructed (Gupta, 2003):

Only if we pursue the truth will we arrive at the most effective means of achieving health.

Only if we pursue EBM will we maximise the likelihood of arriving at the truth.

Therefore, only if we pursue EBM do we arrive at the most effective means of achieving health.

Achieving health is generally regarded as a normative moral good and a case can be made from the three principal branches of normative ethics. Deontological considerations would view health as a moral norm, so we are duty-bound to pursue it in other people and non-human animals (as long as the latter are accepted as subjects of moral consideration). Consequentialism would define health as a morally good outcome to be pursued. Virtue ethics would see achieving health in others as a benevolent act (Alexander & Moore, 2016; Sinnott-Armstrong, 2019; Hursthouse & Pettigrove, 2018). However, the process by which we arrive at health also carries ethical implications. Any methodology that claims to be, or is, better at arriving at this moral good will naturally carry a moral mandate to be used. For EBM/EBVM, this can be stated as follows (Gupta, 2003):

We ought to pursue the most effective means of achieving health.

Only if we pursue EBM do we arrive at the most effective means of achieving health.

Therefore, we ought to pursue EBM.

Rollin has described EBVM as a “requirement”:

A veterinarian has a moral and ethical obligation to provide treatment for which there is good evidence of effectiveness … for any treatment, establishing proof of efficacy as well as potential risks and benefits is an ethical requirement (Ramey & Rollin, 2001).

From here, things quickly become complicated in terms of ethical thinking and its application to EBVM. For the purposes of this discussion, we need not consider anything more taxing than if we accept that achieving health (taken in its broadest sense) is ethically justified, then in terms of practicality we should examine whether EBVM has the necessary qualities to mark it out as the morally obligated way of doing veterinary medicine.

### Justifying EBVM

Is EBVM better? The question can initially be approached in two ways. One, finding an *a priori* reason (knowledge acquired through analysing the concept of EBVM independent of empirical experience) as to why it is a better way, through demonstrating that its epistemological features are superior to other methods of medicine; or two, demonstrating *a posteriori* (knowledge obtained through empirical experience) that its methods produce better results than other forms of medicine.

The preceding discussion has described how EBVM brings nothing particularly new to the epistemological table. It has features of various branches of epistemology, meaning it is vulnerable to existing challenges to these theories. As it is, there is insufficient *a priori* justification for EBVM being better and nor is this likely to obtain. Proponents of EBVM have made a value judgement that objective-as-possible evidence provides the greatest justificatory power to clinical propositions; however, it does not follow (and indeed it would be circular if it did) that the overall approach of EBVM to clinical care is better based on this value-judgement alone. That is, it would be contradictory to the very tenets of EBVM itself if we were to consider the epistemology of EBVM to be superior to other ways of doing veterinary medicine based purely on a subjective value-judgement.

It is also difficult to demonstrate an *a posteriori *justification for superiority. Doubtless there have been several successes in EBM showing superiority to other forms of medicine, especially rationalist and mechanical reasoning (Howick, 2011). It would be inaccurate, however, to say that it has been shown to be always superior or even superior on average.

There are theoretical limits to demonstrating such superiority. Whilst possible tests of EBM/EBVM versus medicine-as-usual have been conceived, they have not been performed, largely due to what is best described as incommensurability between the two sides of the debate (Shahar, 2003). For EBM/EBVM, those who wish to claim they are not superior would be unlikely to accept EBM/EBVM-based methods to test such; equally, EBM/EBVM protagonists would struggle to accept non-EBM/EBVM research criteria/parameters being used to assess EBM/EBVM versus other methods. There is also the question of what to compare EBM/EBVM to, given the myriad of potential ways to perform medicine proposed. In the human field, for instance, they are myriad – narrative-based medicine (Greenhalgh, 1999), context-sensitive medicine (Greenhalgh & Worrall, 1997), tacit knowledge medicine (Malterud, 2001), clinical jazz (Shaughnessy et al., 1998), values-based medicine (Little et al., 2012) and phenomenological-based medicine (Toombs, 2001) have all been suggested, each carrying their own outcome criteria and standards of effectiveness assessment. To an extent, the debate echoes that around alternative therapy effectiveness in veterinary medicine: practitioners maintain they work when assessed within their own paradigm, but not within the constraints of standard scientific study (Ramey & Rollin, 2001).

It appears a long way off before EBVM can demonstrate its superiority epistemologically or empirically, if it ever can. Where does this leave us? Ultimately EBVM cannot conclusively show it is a better way of doing medicine; by extension it cannot satisfy the requirements for being the most ethically sound method of medicine or justify a moral imperative for use. Whilst many believe it is so, it is important to recognise the basis of this belief is not evidence-based. The current justification can only be made from an ethical argument. Such a justification relies ultimately on a value judgement and is not necessarily inherent or entailed by anything EBVM has to offer, so these arguments can conceivably be applied to other ways of doing medicine.

## Closing comments

### Learning from the history of EBM and EBVM

Examining the history of EBM and the issues it has encountered can assist the development of EBVM and help the latter avoid the mistakes of the former. Whilst the histories of EBM and EBVM are intertwined, their futures do not necessarily need to remain so.

Initially EBM Mark I focused primarily on use of evidence, which had two main effects – alienating the medical profession who railed against implied criticism of their methods and appearing to elevate evidence above the other cornerstones of medical practice, clinician expertise, and patient values. Such a focus exposed EBM to strong criticism and resistance. At its inception it failed – and can still be said to be underperforming – in appreciating (and indeed studying or trying to improve) clinician expertise and inclusion of patient values. Evidence is not only as good as how it is produced, but how it fits into individual care. As early critics pointed out, most clinicians were not against using the best evidence for informing care, but that EBM’s apparent early prioritisation of evidence over other aspects somewhat missed the point of practical medicine (Grahame-Smith, 1995).

Evidence-based veterinary medicine as a younger iteration can avoid such issues and so far appears to have mostly done so, though there are some signs that history may be at risk of being repeated. Early enthusiasm for its adoption in regulatory and academic circles led the regulatory body for veterinary surgeons in the UK, the RCVS, tying it to fitness to practise (Jorge & Pfeiffer, 2012) which, 12 years later, appears somewhat premature. More recently, the EBVM manifesto has been produced to allow the profession to “move from a philosophical discussion about what EBVM is – or isn’t – to actually getting on and doing something about it … EBVM is about people and the decisions they make, not the evidence itself” (Veterinary Record, 2020). However, of the 10 manifesto points, 6 are related to synthesis, summary, and promotion of evidence, 2 are about making better shared/informed decisions, 1 is about drug regulation and 1 about future EBVM leaders. Whilst the enthusiasm of getting on with EBVM is laudable, we contend that the focus on evidence in EBVM risks repeating errors documented in EBM.

### Philosophy of EBVM

The philosophy of EBM/EBVM, we have argued, offers no new *a priori* epistemological insights. At its core, it is objectivist in that the more objective the methods used to produce evidence, the more objective and therefore reliable that evidence is; it is therefore vulnerable to criticisms common to other objectivist methodologies. Similarly, it has nothing new to say on the process of induction and IBE for translating study results, applying them to an individual patient et cetera.

However, this does not mean that the applied epistemology of EBVM cannot be improved or seen from a perspective that improves its future application. Most contemporary definitions of EBVM are not just about evidence – so the very labelling of EBVM as EBVM is somewhat oxymoronic. As discussed at length, its definition and application includes clinician expertise and owner circumstances (and possibly patient interests) as key aspects. How can veterinary medicine be practised as evidence-based when it is not based on evidence alone? We contend that the use of such a description that singles out evidence as a base may ultimately be unhelpful in the adoption of EBVM – which has been modest at best (Vandeweerd et al., 2012b) – and how its development is envisaged. There appears to be an ongoing preoccupation with evidence – as described in the EBVM manifesto – at the expense of the other cornerstones of EBVM. Producing more evidence (both basic and clinical) saturates most journals and research foci. Evidence has a role in the foundational epistemological definition of knowledge, of justified true belief (Moser, 2005), by supplying varying degrees of justification depending on its strength; of course, the strengthening such justification is to be valued. However, we argue that the applied epistemology of EBVM should not be simply assumed to be settled and the profession told just to get on with it. The paucity of work, or focus, on the practical integration of evidence with other factors in a clinical encounter highlights the neglect of these parts of the EBVM methodology; without addressing these, it is to be expected that the usefulness of EBVM to the vast majority of veterinarians will remain limited.

Philosophical arguments are important, we argue, because they help examine underlying assumptions and weaknesses in a particular approach. Early critiques of EBM can be seen to have produced two camps, unfairly and unhelpfully delineated by those for or against EBM – the latter described as tied to eminence- or experience-based medicine. We are at pains to prevent such history repeating itself in EBVM. Whilst the founding epistemology of EBVM is beset by problems that are unlikely to be resolved, they may not need to be if the applied epistemology is better characterised and investigated. An appreciation of how there are different ways of doing medicine, that EBVM may need some adjustments, should create a healthy openness of debate around different approaches. It is likely that a combination of these, rather than one alone, will satisfy veterinarian, owner, and patient interests.

### Practical application of EBVM

We have described several issues with the practicalities of applying EBVM to clinical cases: the quality of evidence available both in terms of volume and population bias affecting applicability to unseen patients; how to individualise care using evidence; difference between diagnostic and therapeutic studies; and the commensurability of restricted studies to patients with multimorbidities. We have also outlined the inherent practical and logistical limits of veterinary research with patients, which makes higher-level studies (e.g. RCTs) more difficult, and also mean that the expected yield and usefulness of higher studies may be limited.

The temptation, as described, is to ask for more evidence without direction. We suggest EBVM should avoid acquiring knowledge for knowledge’s sake by the targeted study of relevant, essential research questions likely to benefit the greatest number of patients (those in first opinion practice), producing good quality relevant evidence to inform decision-making. The greater use and refinement of both observational studies and target trial emulations, both more naturally mapping real-world performance of interventions across varied individuals, arguably have potential to achieve this more quickly, cheaply and offering more applicability than do RCTs.

We are of the opinion that clinical expertise is poorly defined and researched in the human, and especially veterinary, field. It appears to refer to a smorgasbord of clinical reasoning, logical thinking, owner and patient interaction, and lots more in between. In EBM it seems to be a catch-all forhow veterinarians act and what they take into consideration during a clinical encounter, with little recognition that all veterinarians do not have the same level of expertise, training, skill, or competence. Yet it remains a cornerstone of EBM/EBVM and attracts little research attention especially compared to clinical conditions. This can be at best be described as surprising – after all, it is the veterinarian who must integrate evidence with other factors to decide on an intervention. In the future, we will attempt to start to correct this imbalance by looking at aspects of clinical decision-making in veterinarians and how these interplay with evidence, the epistemic community, and owner/patient factors. Only by understanding what happens currently can we then aim to better characterise and improve this vitally important area.

Individualisation of the veterinary patient in the clinical encounter has the potential to include their (ascribed) interests or preferences into decision-making. Briefly, this may involve an assessment of the individual characteristics of a patient and assessing these against any proposed intervention. For example, a cat with a fractured femur would likely be best treated with surgical intervention of some sort according to the published evidence. But restricting a feral cat in a hospital for a period of time postsurgery is, on balance, probably not in that cat’s best interests despite any future – possible – benefits, so tailoring the approach may mean that amputation and early release is preferable for the feral cat as opposed to the best treatment for an owned cat that shows no fear of humans. We believe that a patient-centred, phenomenological approach to care where the individual patient’s interests are considered alongside the published evidence is possible (Mills, 2021). Such care is informed by evidence but is unlikely to be determined by evidence, at least not in isolation. It has some similarities with what has been described as contextualised care, a term that has gained traction lately (Hanaghan, 2022).

### Ethics of EBVM approach

As we have discussed, it cannot be said that EBVM as it currently exists can demonstrate *a priori *or *a posteriori* superiority as a clinical approach; the ethical imperative for use of EBVM does not as yet obtain. Indeed, it may never do so, given issues described with testing it versus other approaches. As such, requirements to use it and aligning it to fitness to practise cannot be justified. However, such lack of ethical imperative does not mean that we are arguing against the central tenets of EBVM: good quality evidence should inform clinical decisions; veterinarian expertise is vital in applying the evidence, as are owner and patient circumstances. Rather, we argue that an ethical approach to veterinary medicine would not simply be evidence-based but evidence-informed.

### How to do EBVM better

We have provided extensive commentary and critique of EBVM 20 years after its inception. It is our opinion that it remains a nascent movement and its future direction can be altered in order to better meet the needs of veterinarians, owners, and patients; we believe there is a strong moral argument that it should be. We draw these conclusion from both our experiences as practising veterinary surgeons and longstanding interest and research in epistemology and applied ethics; most importantly, we wish to see the continued, targeted and effective progression of veterinary care, informed by evidence but individualised to each patient.

Ultimately, what do we believe a better EBVM would look like? We have discussed improvements above and summarise them here for clarity. Improving the quality of research by employing methods that make results more applicable to the majority of veterinary patients, that is, those in first opinion practice; greater focus on veterinarian expertise, including interrogating the process of decision-making, how evidence and owner and patient circumstances may be appropriately integrated into a clinical decision and how these processes may be made more reliable by giving sufficient consideration to stakeholders and evidence; better individualisation of veterinary care to patients; reappraising clinical veterinary practice as not simply evidence-based but evidence-informed; understanding and re-appraising the social (professional) epistemological value of experience within veterinary medicine. We appreciate these changes may appear challenging to implement but that does not diminish their importance. In future work we intend to expand on these improvements, providing more detail than is possible here.

[1] Carried out by the first author for the purposes of this article

## About the authors

David Mills has been a practising first-opinion veterinary surgeon for over 17 years and has previously provided commentary on EBVM and its ethical aspects. His doctoral thesis looked at the practical, ethical, and future aspects of EBVM. He has undertaken further qualifications at UK certificate level in cardiology; animal welfare science, ethics and law; small animal surgery; emergency and critical care; and small animal neurology. He currently splits his time between first opinion and cardiology referral practice.

Michael Reiss is Professor of Science Education at University College London, a member of the Nuffield Council on Bioethics, and a Fellow of the Academy of Social Sciences. He was the ethicist on the Advisory Committee on Novel Foods and Processes, the ethicist on the Farm Animal Welfare Council, chaired EuropaBio’s External Advisory Group on Ethics, and spent 18 months as Specialist Advisor to the House of Lords Select Committee on the Use of Animals in Scientific Procedures.

Madeleine Campbell is Professor of Veterinary Ethics at Nottingham University School of Veterinary Medicine and Science and a RCVS and European EBVS Specialist in Animal Welfare Science, Ethics and Law. She currently chairs the independent Animal Welfare Committee which advises Defra and the Scottish and Welsh governments, and through a number of appointments provides advice to various organisations including the Greyhound Board of Great Britain, the British Equestrian Federation, and the British Horseracing Authority. Her clinical background is in veterinary reproduction.

## ORCiD

David Mills: https://orcid.org/0009-0009-3483-9799

Michael Reiss: https://orcid.org/0000-0003-1207-4229

Madeleine Campbell: https://orcid.org/0000-0003-1123-5828

## Conflict of Interest

The author declares no conflicts of interest.
